# The consequences of migration and displacement for mental health: A qualitative study of river nomadic communities

**DOI:** 10.1017/gmh.2026.10230

**Published:** 2026-05-26

**Authors:** Md. Monirul Islam, Nafiul Muid, Rajiv Acherjee, Anika Tasnim, Mezabahnur Masum

**Affiliations:** 1 Economics, Jatiya Kabi Kazi Nazrul Islam University, Bangladesh; 2 Islamic University, Bangladesh; 3 Department of Social Services (DSS), Bangladesh; 4 Khulna University, Bangladesh; 5 Independent Researcher

**Keywords:** river nomads, environmental displacement, mental health, psychosocial resilience, climate migration, Bangladesh

## Abstract

This study explores the psychosocial consequences of recurrent environmental displacement among river-nomadic (Bede) communities in Bangladesh, emphasizing how cyclical mobility, ecological degradation, and social marginalization shape mental health and resilience. A qualitative phenomenological approach was adopted, analyzing twelve in-depth interviews and one focus group discussion from three Jamuna River settlements (2025 monsoon season) via NVivo 14. We identified six themes: income-driven relocation, environmental hazards as triggers, cumulative physical and emotional tolls, negotiation/reputation as safety mechanisms, culturally rooted coping, and aspirations for stability. Participants described chronic uncertainty alongside strong communal solidarity and faith-based coping. Findings highlight that displacement among river nomads is a continuous, multifaceted psychosocial exposure rather than an isolated event. Sustainable interventions should integrate mobile, community-based mental health and psychosocial support (MHPSS) services with environmentally informed livelihood programs and inclusive governance. This research contributes empirical evidence to the discourse on climate-related migration, emphasizing the need for culturally grounded, mobility-sensitive approaches for marginalized riverine populations.

## Graphical abstract

**Figure 1:**
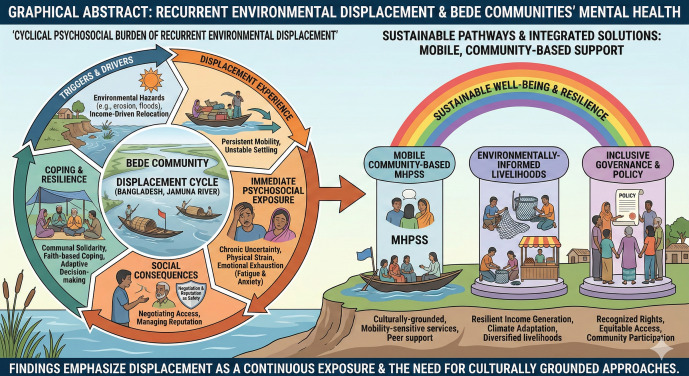

## Impact statement

River-nomadic (Bede) populations in Bangladesh endure a displacement–displacement loop, which is caused by flooding, riverbank erosion, as well as declining ecological livelihoods. These communities are in near-perpetual motion, unlike refugees who flee one event, and as such, they move several times in a year, which makes their mental health needs mostly invisible to traditional, facility-based health systems. This article gives the initial qualitative, phenomenological report of how these sustained displacement conditions shape psychological anguish, coping and endurance among Bede communities residing along the Jamuna River.

The results prove that the state of chronic nomadic displacement is a prolonged psychosocial exposure, rather than a crisis. It produces cumulative anxiety, physical fatigue and identity disruption, as well as causing culturally encoded coping in communal solidarity, faith-based practices and reputation management. These lessons have a direct implication on global mental health programming in climate-vulnerable, low- and middle-income countries.

In particular, this study provides evidence that mental health and psychosocial support (MHPSS) services need to be formulated in such a way that they can be cellular, community-based and culturally based, i.e. not clinic-based models that cannot be offered to the mobile population. The research contributes to the developing literature on climate change and mental health by making a population considered marginalized the focus of the study, whose experiences have not been often recorded in broad-based epidemiological studies. This article recommends that policymakers, NGOs and health planners should implement mobility-sensitive, rights-based approaches to riverine and displaced communities in Bangladesh and similar deltaic situations in South and Southeast Asia by addressing institutional inadequacies in the provision of livelihood security and basic services.

## Introduction

Global and regional scholarship increasingly recognizes migration and displacement as multidimensional processes shaped by environmental change, livelihood precarity and socio-political marginalization, with direct implications for both physical and psychosocial well-being (Bhuiyan et al., 2017; Haque et al., 2022; Rahman et al., 2022). In low-lying deltaic contexts such as Bangladesh, riverine processes – particularly riverbank erosion, seasonal flooding and alterations in aquatic ecosystems – produce recurrent, localized displacement that is cyclical and survival-oriented rather than linear or permanent (Bhuiyan et al., 2017; Shawon et al., 2022). Empirical studies demonstrate how riverbank erosion and land loss precipitate repeated household relocations, fragment social networks and erode economic security, amplifying vulnerability to subsequent hazards and constraining adaptive choices (Bhuiyan et al., 2017; Rahman et al., 2022). Concurrent declines in riverine resources – such as fluctuating fish stocks, habitat degradation and extractive activities like sand mining – further reduce the ecological carrying capacity for traditional livelihoods and intensify competition over dwindling natural resources (Al-Mamun et al., 2023; Yoshimura et al., 2023). Collectively, these environmental stressors generate a landscape in which mobility functions as a recurrent livelihood strategy for survival rather than a voluntary pursuit of economic opportunity (Shawon et al., 2022).

River-dwelling or “river nomadic” communities in Bangladesh – commonly referred to as *Bede*, *Bedeni*, or boat-dwelling groups – occupy a distinct socioecological niche defined by itinerant livelihoods, artisanal occupations (such as fishing, transient trade, folk medicine and performance) and a longstanding history of social marginalization (Shawon et al., 2022; Yoshimura et al., 2023). Estimates are available that indicate that psychological issues such as depression, anxiety and post-traumatic stress disorder are overrepresented in those displaced by rivers in Bangladesh, and rates of clinically significant symptoms have been reported ranging between 20% and 40% among communities affected by floods (Hossain et al., 2021; Karim et al., 2025). Irrespective of such numbers, the river-nomadic populations are still not well represented in epidemiological surveys, which prompts the necessity of the qualitative study of their experiences. Ethnographic and health service studies highlight the Bedes’ precarious access to healthcare, low social acceptance in settled communities and the ongoing pressures of sedentarization that threaten intergenerational occupational identities (Yoshimura et al., 2023). These processes interact with environmental degradation: as river courses shift and ecological productivity declines, nomadic fishers and boat-dwelling families experience intensified livelihood insecurity, compelling many into frequent relocations or forced settlement in marginal urban and peri-urban zones with limited rights and social protection (Bhuiyan et al., 2017; Shawon et al., 2022). Furthermore, macro-scale processes of territorialization, urban expansion and regulatory contestation over riparian zones have curtailed river access and exacerbated dispossession among mobile riverine populations (Huq and Azaz, 2024).

The cyclical and often involuntary nature of displacement among river nomads produces distinct psychosocial stressors. Recurrent habitat loss, disrupted livelihoods and the weakening of social and cultural anchors contribute to chronic stress, uncertainty and the erosion of identity tied to place and occupation (Shawon et al., 2022; Ara et al., 2025). Research from Bangladesh and comparable riverine settings highlights the psychological burden associated with repeated loss – of homes, boats and customary fishing grounds – alongside the social stigma and barriers to formal health services that impede help-seeking for emotional distress (Haque et al., 2022; Shawon et al., 2022). Gendered dimensions of vulnerability, including constrained female agency and unequal exposure to livelihood shocks, further compound psychosocial risks within river-dependent communities (Kamal et al., 2018; Ara et al., 2025). Although mobility is sometimes framed as resilience when it expands livelihood opportunities, for river nomads, it often undermines social capital and coping resources, producing cumulative mental health consequences distinct from single-event displacement (Bhuiyan et al., 2017; Shawon et al., 2022).

Despite growing evidence that environmental pressures and recurrent displacement shape material vulnerability in Bangladesh, qualitative and phenomenological inquiry into how chronic, cyclical displacement affects the psychosocial well-being of river nomadic communities remains limited. Existing studies have primarily examined material outcomes – such as land loss, declining fisheries and barriers to service access for *Bede* and riverine fishers – yet few have explored in depth the lived experiences of chronic displacement: the meanings attached to mobility, the subjective feelings of uncertainty and identity disruption, and the culturally specific idioms of distress and coping that inform mental health outcomes (Nichter, 1981; Shawon et al., 2022; Yoshimura et al., 2023). Where research has addressed *Bede* communities, the focus has often been on discrete biomedical issues or structural exclusion rather than on the phenomenological dimensions of psychosocial suffering and resilience (Haque et al., 2022; Yoshimura et al., 2023). Similarly, migration scholarship concerning dispossession and urban nomadism has highlighted governance and economic drivers but rarely conceptualized prolonged, cyclical displacement as a sustained psychosocial exposure in riverine contexts (Bhuiyan et al., 2017; Mondal, 2024).

Understanding these lived experiences is essential for developing culturally appropriate and context-sensitive mental health and psychosocial support (MHPSS) interventions. Qualitative and interpretive approaches can uncover local explanatory models of distress, help-seeking practices and collective resilience strategies that quantitative frameworks often overlook, thus informing interventions that are both feasible and acceptable for mobile riverine communities (Kleinman, 1980; Kamal et al., 2018; Ara et al., 2025). Moreover, integrating environmental and governance dimensions – such as the impacts of sand mining, territorialization and fisheries decline – into mental health programming is crucial where ecological degradation acts as a proximal determinant of psychosocial risk (Chowdhury et al., 2019; Huq and Azaz, 2024). Without such grounded knowledge, policy and service responses risk perpetuating exclusion by relying on stationary, facility-based models of care unsuited to nomadic populations whose mobility, mistrust of formal institutions and stigmatized identities constrain service engagement (Shawon et al., 2022; Yoshimura et al., 2023).

This study addresses these gaps through a qualitative phenomenological investigation of the psychosocial consequences of migration and displacement among river nomadic communities in Bangladesh. By centering narratives of livelihood change, environmental precarity and mental distress within the broader processes of ecological degradation and social marginalization, the study contributes new empirical insights to the fields of migration studies, global mental health and disaster risk reduction. It advances conceptual understanding of resilience versus chronic vulnerability among mobile river populations and generates evidence to inform culturally congruent, mobility-sensitive mental health interventions and policy responses in Bangladesh and comparable deltaic settings (Bhuiyan et al., 2017; Shawon et al., 2022; Yoshimura et al., 2023; Ara et al., 2025).

## Research objectives

Grounded in the preceding analysis and knowledge gaps, this study was guided by three interrelated objectives designed to capture the lived experience and psychosocial dimensions of displacement among river nomads in Bangladesh:

1. To understand how repeated displacement affects the mental health and well-being of river nomadic individuals and families.
2. To explore the coping strategies and resilience mechanisms employed in response to chronic mobility and social marginalization.
3. To generate phenomenological insights that inform culturally relevant and context-sensitive mental health support for mobile communities.

## Methodology

### Research design and philosophical orientation

This study adopted a qualitative phenomenological design within a constructivist–interpretivist paradigm, aiming to explore the lived experiences of displacement, uncertainty and psychosocial distress among *Bede* (river nomadic) communities residing along the Jamuna River in Tangail District, Bangladesh. The phenomenological approach was chosen to capture participants’ subjective meanings and emotional realities as they navigate chronic environmental change and cyclical displacement (Creswell and Poth, 2018). Under the constructivist paradigm, knowledge is understood as co-created through dialogue between researcher and participant, acknowledging that mental health experiences are socially and contextually constructed. In particular, this article follows the reflexive thematic analysis (RTA) conceptualized by Braun and Clarke (2006, 2021) according to which the researcher will be an active thematic creator, but not a passive discoverer of patterns in the data (Braun and Clarke, 2021; Morriss, 2024). In line with this, all through this manuscript, the themes are explained as something created in the process of the researcher interaction with the data, rather than a preexisting pattern that was created by the data.

Researcher Positionality: The first author, who conducted primary analysis and data collection, is a Bangladeshi scholar with previous experience of working in the field doing research on riverine and marginalized neighborhoods. Although knowledge of the local setting facilitated the development of rapport and the ability to communicate in the same language as locals, the research team concedes that the insider-next-door role can have an influence on the interpretive process. Reflexivity Reflexive journaling was undertaken during data collection and analysis, co-author peer debriefing and analytic decision audit-trailing. The research team did not identify themselves with Bede participants in terms of ethnicity, occupation and livelihood, and this could have played a role in power dynamics in the course of interviews. The latter reflexive considerations align with the epistemological assumptions of constructivist–interpretive qualitative inquiry (Creswell and Poth, 2018).

### Study setting

The study was conducted in Tangail District, a central Bangladeshi region traversed by the Jamuna River, one of the most erosion-prone river systems in South Asia. The selected field sites – Jugiyar Char Bede Bohor, Palima Bede Bohor and Char Nikrail Bede Bohor – are small riverine settlements (locally called *bohor*) where nomadic *Bede* families reside temporarily on boats or semi-permanent char islands. These locations were chosen purposively because they represent different ecological and social contexts of displacement, varying in flood exposure, riverbank erosion and access to nearby markets or services. The communities depend primarily on itinerant occupations such as snake charming (*sapure*), traditional healing (*bajigor*) and small-scale trading, all of which are increasingly threatened by environmental instability and social exclusion.

### Participants and sampling strategy

A total of 12 participants were interviewed for this study, comprising 10 men and 2 women aged between 22 and 70 years. The participants included *sardars* (community leaders), *sapures* (snake charmers), *bajigors* (magician-healers) and the wife of a *sardar*, *bedeni* (female sapure), representing both leadership and occupational diversity within the *Bede* community.

Given the social marginalization and mobility of this group, recruitment required careful cultural sensitivity and flexibility. A two-stage sampling approach, purposive followed by snowball sampling, was adopted. Initially, one participant from each *bohor* was selected purposively based on visibility, leadership and willingness to engage. These initial contacts later facilitated introductions to additional participants through snowball referrals, enabling trust-based access to a population typically cautious of outsiders.

Recruitment was challenging because *Bede* respondents were often reluctant to discuss personal or emotional topics, especially with external researchers. Establishing rapport took multiple informal visits and reassurance regarding confidentiality. Although the sample size (*n* = 12) is modest, it is methodologically justified within a reflexive phenomenological framework that prioritizes *depth over breadth* (Creswell and Poth, 2018). The goal was to achieve information saturation rather than numerical representation. By the final interviews, thematic repetition was evident across participants, indicating adequate saturation of core meanings and patterns. This is also supported by the empirical and conceptual evidence of the qualitative literature: Guest et al. (2006) have shown that data saturation is generally achieved in semi-structured qualitative research with a sample of 12 interviews, whereas Malterud et al. (2016) have provided an information power model according to which small, information-rich samples with a specific, purposive population may be sufficiently large.

Furthermore, the difficulty of access, cultural sensitivities and the mobile nature of *Bede* life make larger samples impractical and ethically questionable. In qualitative inquiry with hard-to-reach or vulnerable groups, smaller, information-rich samples are widely accepted (O’Brien et al., 2014; Patton, 2015).

### Data collection

Data were collected during the mid-monsoon season of 2025, when the Jamuna River’s rising waters, erosion and flooding made migration inevitable for *Bede* families. The first and third authors conducted all fieldwork and data collection. Conducting fieldwork during this period allowed the researchers to capture participants’ real-time experiences of displacement, anxiety and adaptation. Semi-structured, in-depth interviews were carried out across three settlements – Jugiyar Char, Palima and Char Nikrail Bede Bohor – using a flexible interview guide focused on migration history, mental well-being and coping strategies. Each interview lasted 45–75 min and was conducted in the participants’ local dialect, often beside their boats or temporary riverbank shelters. With participants’ consent, all interviews were audio-recorded, transcribed verbatim in Bangla and translated into English. Field notes were maintained to record observations and reflections.

The focus group discussion (FGD) was carried out in combination with the individual interviews to come up with complementary data. Although the in-depth interviews were intended to yield personal, biographical accounts of displacement and distress, the FGD gave a platform for collective discussion and enabled members to support, refute or elaborate more on themes brought out in individual accounts. This phenomenological approach to data collection is in line with a mixed approach of such research as it aims to identify both personal lived experience and collective meaning-making in a community (Creswell and Poth, 2018). Collecting data during the monsoon enhanced the authenticity and phenomenological depth of participants’ accounts, as their struggles and resilience were unfolding in real time.

### Data analysis

Data were analyzed using RTA, following the six-phase framework of Braun and Clarke (2006, 2021). Primary coding and analysis were led by the first and second authors, with coding support from the fourth author. All Bangla transcripts were first read repeatedly to ensure familiarization with the data, followed by manual and NVivo 14-assisted coding. Codes were generated inductively to remain grounded in participants’ lived experiences. These codes were then clustered into broader categories and refined into overarching themes that captured the psychosocial consequences of displacement and coping strategies among *Bede* communities. The analytic process was iterative and reflexive, involving constant comparison between transcripts, field notes and emerging interpretations. Preliminary themes were reviewed several times to ensure coherence, internal consistency and distinctiveness. In keeping with Braun and Clarke’s (2021, 2023) guidance on reflexive TA, themes were treated as constructions actively developed by the research team through systematic engagement with the data, rather than as patterns that objectively emerge from it (Morriss, 2024). The researcher maintained an audit trail documenting analytic decisions and reflections throughout the process. Six major themes were constructed, representing the complex interrelation between environmental displacement, livelihood disruption, mental distress and resilience among river nomads. This study was conducted in accordance with the Consolidated Criteria for Reporting Qualitative Research (COREQ) checklist (Tong et al., 2007), which is submitted as a supplemental file.

### Ethical considerations

The procedures used in ethics were based on the principles of voluntary participation, informed consent in written form, protection of privacy of the participant and minimization of harm related to sensitive disclosures. Data collection was done with informed consent, which was in writing; there was no withdrawal penalty at any time. No clinical intervention was done.

## Findings

### Participant characteristics

The study engaged twelve participants from Bede (river nomadic) communities residing along the Jamuna River in Tangail District, Bangladesh. Participants were drawn from three Bede Bohor settlements, Jugiyar Char, Palima and Char Nikrail, representing diverse ecological and social contexts of displacement. The group consisted of ten men and two women, aged between 22 and 70 years, reflecting intergenerational perspectives within the community. Occupationally, participants included sardars (community leaders), sapures (snake charmers) and a bajigor (magician-healer), representing both leadership and traditional livelihood roles central to Bede identity. Table 1 summarizes the demographic characteristics of the interview participants, while Table 2 summarizes the demographic characteristics of the FGD respondents.

**Table 1.** Demographic characteristics of interview participants Table 1. long description.

Participant ID	Sex	Age (Years)	Occupation / Social role
Interview 1	Male	70	Sardar (community leader)
Interview 2	Male	32	Sapure (snake charmer)
Interview 3	Male	60	Bajigor (magician and healer)
Interview 4	Male	31	Sapure (snake charmer)
Interview 5	Male	47	Sapure (snake charmer)
Interview 6	Female	56	Sardar’s wife
Interview 7	Female	25	Sapure (bedeni) (snake charmer)

**Table 2.** Demographic characteristics of FGD respondents Table 2. long description.

Respondent ID	Sex	Age (Years)	Occupation / Social role
Respondent 1	Male	35	Sapure
Respondent 2	Male	42	Sapure
Respondent 3	Male	46	Sapure
Respondent 4	Male	58	Bajigor
Respondent 5	Male	22	Unemployed

**Table 3.** Summary of key findings Table 3. long description.

Theme	Core insight	Psychosocial implication
1. Income-driven relocation	Mobility is a planned survival strategy guided by shifting livelihood opportunities.	Economic precarity fosters adaptive decision-making but sustains chronic instability.
2. Environmental hazards	Floods, erosion and storms force rapid displacement and recurrent loss.	Environmental stress translates into cumulative anxiety and trauma.
3. Physical and emotional toll	Each relocation imposes acute physical exhaustion and emotional distress, expressed through culturally specific idioms of suffering.	Cumulative distress requires recognition of local idioms and culturally sensitive screening beyond Western clinical instruments.
4. Social negotiation and reputation	Settling safely depends on negotiating acceptance with locals and maintaining social reputation.	Social capital becomes a protective but fragile buffer against exclusion.
5. Culturally rooted coping and resilience	Communities actively cope through faith-based practices, communal mutual aid and adaptive meaning-making rooted in cultural explanatory models.	Indigenous resilience strategies provide an essential foundation for culturally adapted MHPSS co-design and community-based delivery.
6. Aspirations for stability	Participants desire secure housing, steady income and institutional recognition.	Highlights unmet policy needs and the psychosocial cost of systemic neglect.

### Thematic analysis

We identified six interconnected themes through RTA: economic drivers of mobility, environmental vulnerability, physical and psychological burden, social negotiation, health and coping strategies and aspirations for stability.

#### Theme 1: Income-driven relocation – adaptive strategies for survival

Migration in these communities is largely driven by the need to survive. Moves are deliberate and carefully planned, guided by where and when opportunities to earn a living arise. Their settlements are influenced by the kind of economic activities they engage in, such as entertainment performances or traditional therapies, which dictate both location and length of stay.

As the 70-year-old Bede sardar noted, “Sometimes we stay a month in one place, then move on when people no longer come to our shows; we follow where work and income are possible.”

The nomadic communities make relocation decisions with a keen understanding of local economic dynamics. They are aware of how demand for their services fluctuates over time and how social reputation influences their ability to return and settle in familiar areas. This careful consideration allows them to plan movements strategically, ensuring that each relocation maximizes both safety and livelihood opportunities.

The sardar’s wife explained, “If people remember us from before and we helped them, they welcome us again; it helps us earn safely and consistently.”

The participants’ stories reveal a nuanced grasp of how local economies signal opportunities. Their decisions about when and where to move are shaped by past experiences and careful observation of community behavior, helping families maximize earnings while steering clear of areas with little potential. Mobility, therefore, is a deliberate adaptive strategy rather than a mere reaction to circumstance. Economic pressures also interact with other vulnerabilities, such as food insecurity and the unpredictability of local markets, reinforcing the necessity of constant movement.

#### Theme 2: Environmental hazards as drivers of forced migration

Natural problems like floods, riverbank erosion and storms cause people to move right away and also shape how they move over time. The participants said they often lose their homes and belongings, which forces them to move quickly to a new place.

As one 32-year-old sapure participant explained, “When the river rises, everything is washed away. We have to pack in the middle of the night and move to another place.”

These events cause not just loss of things but also stress and practical problems, because the whole community has to take apart and move their homes together.

Another 60-year-old bajigor reflected, “We rebuild our tents again and again, but every time it is harder, and the children get scared during storms.”

This problem becomes even bigger because the rivers and canals in Bangladesh are changing. In the past, many communities lived and worked on boats, depending on water for their jobs. Now, some canals and river channels are drying up, so they cannot live on the water anymore. This forces them to move to land and change the way they build their homes and earn their living. Environmental problems act as both limits and drivers for how people adapt to life and work. They show how closely nature and livelihoods are connected, and how these challenges influence when, where and how communities move.

#### Theme 3: The cumulative physical and emotional toll of recurrent displacement

Repeated relocation imposes a compounding physical and psychological burden on Bede individuals and families. Each displacement episode demands the dismantling, transport and reconstruction of temporary shelters – labor-intensive work that falls disproportionately on women, who simultaneously manage childcare, cooking and domestic responsibilities across unsettled terrain. Over successive relocations, this effort accumulates into chronic physical exhaustion that participants experience as inseparable from their nomadic condition.

The sardarni (wife of sardar/leader) described the immediate physical toll of each move: “Every time we move, I carry water, cook, and help set up the tents while looking after the children. By evening, my back aches, and I feel completely drained.”

The repeated work increases exhaustion, and children and older adults experience extra risks and hardships.

A 25-year-old Bedeni highlighted the distress experienced by the most vulnerable members of the community: “The children cry when we have to leave quickly, and the older ones get tired walking with us. It’s exhausting for everyone.”

Participants also articulated this distress through culturally rooted idioms (Nichter, 1981). Many described a pervasive emotional heaviness they called “moner koshto” (heartache or suffering of the mind) and “chinta” (worry/rumination), which they distinguished from physical illness but understood as a consequence of unrelenting uncertainty. These expressions clustered around fears of losing livelihood, being unwelcome in new places, and failing to protect their children. A 47-year-old Sapure articulated this sleepless preoccupation: “Every move makes me anxious about food, my children’s schooling, and whether we’ll have a safe place to sleep. Sometimes I can’t even sleep at night thinking about it.” This sleeplessness and preoccupation align with symptom-level presentations of generalized anxiety and psychological distress, even in the absence of formal clinical diagnosis. Collectively, the physical exhaustion and emotional suffering documented in this theme represent the immediate, episode-level burden that each displacement event imposes on Bede individuals and families.

#### Theme 4: Leveraging reputation and networks for safe relocation

Moving to a new place is not just about finding land; it requires talking and negotiating with local people. The participants explained that they always reach out to local authorities, landowners and respected community members to get permission before settling. Building good relationships and using social connections is very important to make sure they can stay safe and without trouble.

As the sardar explained, “Before we stay anywhere, we first talk to the landowner and the local youth. If they agree, we set up; if not, we simply move elsewhere.”

Whether they were welcomed in a new place often depended on their past behavior and reputation. Families who had helped local people before or stayed respectfully were more likely to be treated kindly when they returned. These stories show that moving from place to place is not just about travel; it involves careful planning and building relationships.

Another Sapure described the social calculus involved: “When we arrive somewhere new, people watch us carefully for the first few days. If we behave well and do not cause trouble, they start to trust us. That trust is what keeps us safe.”

The communities do not just wait for locals to accept them; they actively negotiate, help each other and maintain a good reputation to stay safe and secure access to resources. When people in a new area are suspicious or unfriendly, moving to another place is usually the safest choice. Strong bonds within the nomadic groups help them handle uncertainty from outside. Families support each other by sharing food, money and care, which makes it easier to cope with challenges in new places. This adaptive strategy allows them to navigate complex social landscapes while minimizing conflict and maintaining continuity in their livelihoods.

#### Theme 5: Culturally rooted coping and adaptive resilience strategies

Despite the physical and emotional toll documented in the preceding theme, Bede communities have developed a rich repertoire of culturally embedded coping strategies through which they navigate chronic displacement. Rather than passive victims of structural hardship, participants demonstrated active, purposive resilience – drawing on spiritual resources, communal solidarity and adaptive meaning-making to sustain well-being across successive relocations.

When participants reflected on how they interpreted and made sense of their chronic distress, their accounts aligned closely with Kleinman’s (1980) concept of explanatory models of illness. Rather than attributing their suffering to discrete events, they explained their mental and physical condition as the product of accumulating pressure: the relentless movement, absence of rest and inability to plan ahead. A number of participants described being *achol* (restless/unsettled) not as a passing emotional state but as a near-permanent condition of being, inseparable from their nomadic lives. This explanatory framing is significant: it locates the source of distress in the structure of nomadic life rather than in individual pathology, and points toward structural as well as psychosocial solutions. Parents were particularly attuned to the effects of displacement on their children, noting how constant relocation disrupts schooling, severs friendships and erodes children’s sense of belonging – compounding their own sense of responsibility and guilt.

One sardar articulated this collective orientation toward endurance: “We cannot change the river, and we cannot stop moving. So we hold on to each other and to God. That is how we survive.”

Faith and spiritual practices constituted the primary individual-level coping resource. Participants described saying prayers, making personal vows and performing small rituals as means of restoring a sense of calm and agency during uncertain transitions. These practices were not merely consolatory; they functioned as active mechanisms of emotional regulation, providing participants with a sense of continuity and divine protection that partially offset the instability of their material conditions. Communal solidarity was the primary collective-level coping strategy. Families shared food, pooled resources and organized informal care for sick or vulnerable members. This mutual aid network operated as an informal social safety net that mitigated the immediate impact of hardship when no formal support was available. A 35-year-old Sapure reflected on the centrality of this communal ethic: “When someone in our group is struggling, we all feel it. We share what we have – food, money, care. That is how we keep going.” Together, these spiritual, communal and adaptive coping strategies constitute a coherent, culturally grounded system of resilience. They do not eliminate the distress documented in Theme 3, but they represent the community’s active and purposive response to it – one that external MHPSS programming must understand, respect and build upon rather than supplant.

#### Theme 6: Aspirations for stability and the need for institutional support

Even though they show great resilience, participants strongly wish for stable homes, steady income and support from institutions. Living in temporary shelters with uncertain earnings causes ongoing stress and makes it hard to focus on children’s education, health and social life.

The 60-year-old bajigor described, “If we had a safe place to stay and a steady way to earn, life would be so much easier. Right now, we never know where we will sleep tomorrow.”

Another 46-year-old added on the absence of external aid: “Many government officials and NGOs have visited, made promises, but nothing has ever reached us. We are left to manage on our own.”

Participants report that support from the government or NGOs is very limited, irregular, or completely lacking, which increases their vulnerability and makes their situation even more difficult. Participants emphasize that even small interventions could have transformative effects.

As one 35-year-old sapure noted, “We don’t need much, just a small plot for living, some help with work, and access to medicine and schools. That alone would change everything for our families.”

The participants’ accounts show a clear understanding of two realities: they have built strong adaptive skills to survive on their own, yet external support from structured programs could greatly enhance their living conditions. Even basic provisions, such as secure housing, reliable income and access to healthcare, would significantly lessen the physical, social and mental burdens caused by constant relocation. Participants frame these needs not as entitlements but as prerequisites for dignity, safety, and the capacity to sustain their livelihoods. Their accounts highlight the tension between resilient coping and systemic neglect, revealing that sustainable support would amplify their agency rather than replace it.

## Discussion

This qualitative study, conducted among Bede (river-nomadic) communities along the Jamuna River, constructed six interlocking themes that explain how cyclical displacement shapes mental health and everyday life: (1) income-driven relocation as a deliberate survival strategy; (2) environmental hazards (flooding, erosion, storms) as acute triggers of forced movement; (3) the cumulative physical and emotional toll of recurrent displacement, expressed through locally specific idioms of distress; (4) reliance on reputation and local negotiations to secure temporary settlement; (5) culturally rooted coping and adaptive resilience strategies, including faith-based practices, communal mutual aid and explanatory model-informed meaning-making; and (6) aspirations for stability coupled with persistent gaps in governmental and NGO support. Together, these themes portray displacement not as a one-off event but as an ongoing exposure that erodes social anchors and intensifies psychosocial vulnerability while simultaneously eliciting culturally embedded resilience.

Our findings are consonant with Bangladesh-focused evidence showing that riverbank erosion and flood-related relocation are associated with elevated symptoms of depression, anxiety and stress, and that these effects persist where livelihoods and services remain unstable (Hossain et al., 2021; Karim et al., 2025). Notably, our research builds on this body of evidence by showing that this distress in populations of river-nomads is not just a reaction to discrete episodes of flood, but a cumulative, psychosocial exposure to its structural circumstances and consequences of nomadic life. The distress words used by the participants, “moner koshto,” “chinta,” and “achol,” correspond to, but do not match, Western clinical levels of anxiety and depression, which underlines the importance of culturally specific screening and support instruments in this situation (Nichter, 1981). They also extend regional literature that frames riverine mobility as cyclical and survival-oriented rather than linear, with environmental degradation and resource competition as proximal drivers of repeated moves (Haque et al., 2022; Rahman et al., 2022; Shawon et al., 2022). The salience of identity loss – disruption of boat-based living, traditional fishing/performative occupations and gendered burdens of care – echoes accounts of Bede marginalization and barriers to formal care (Yoshimura et al., 2023).

At a broader level, our results corroborate scholarship conceptualizing displacement as a *processual* exposure with cumulative mental-health consequences rather than a discrete shock. Although the literature on climate-mental health antecedently has reached out to anticipatory anxiety and ecological grief (Cunsolo and Ellis, 2018), our results are based on first-hand accounts of the participants, which ground the theorized constructs of anticipatory anxiety and ecological grief on the lived experience of the Bede communities. Those who talked about mourning the loss of river channels and fishing grounds through the use of emotionally charged words matched the Cunsolo and Ellis model of ecological grief, the grieving of places that were part of the identity and livelihood. It is a new empirical entry: instead of assuming that such grief can be ascertained based on climatic data, we record its expression in the subjective form of accounts of loss and displacement stories. Systematic and scoping reviews likewise document consistent associations between climate-related hazards and common mental disorders, with disproportionate impacts on socially marginalized groups, conditions closely mirroring the Bede context (Hayes et al., 2018; Cianconi et al., 2020; Crane et al., 2022).

The community strategies highlighted here – leveraging reputation, negotiating access with local elites and sharing food, cash and caregiving – map onto the disaster-resilience literature on bonding, bridging and linking social capital. Studies after the Nepal earthquake show that such social capital channels are decisive for access to aid, information and collective action (Panday et al., 2021; Rayamajhee and Bohara, 2021). Classic syntheses similarly argue that social infrastructure can be as critical as physical infrastructure for recovery – an observation reflected in participants’ emphasis on trusted relations and “being known” as prerequisites for safe settlement and steady earnings (Aldrich and Meyer, 2015). Although the literature on climate-mental health antecedently has reached out to anticipatory anxiety and ecological grief (Cunsolo and Ellis, 2018), our results are based on the first-hand accounts of the participants, which ground the theorized constructs of anticipatory anxiety and ecological grief on the lived experience of the Bede communities. Those who talked about mourning the loss of river channels and fishing grounds through the use of emotionally charged words matched the Cunsolo and Ellis model of ecological grief, the grieving of places that were part of the identity and livelihood. It is a new empirical entry: instead of assuming that such grief can be ascertained based on climatic data, we record its expression in the subjective form of account of loss and displacement stories.

Participants’ accounts of their needs reveal an important structural distinction that has direct implications for MHPSS programming. The modest, predictable supports they described – secure land plots, facilitation of livelihood activities, access to medicines and schooling for children – are best understood as structural preconditions rather than MHPSS interventions per se. These material conditions constitute the foundational layer of safety and predictability upon which psychosocial engagement becomes meaningful and feasible. As Heim and Kohrt (2019) argue, the cultural adaptation of psychological interventions must begin with a careful assessment of the structural and social context in which mental distress is embedded; without addressing material precarity, MHPSS delivery risks irrelevance for populations whose primary preoccupation is daily survival. Once these structural supports are in place, or integrated into a layered package, MHPSS interventions can be designed to be community-based, task-shared and mobility-sensitive. Sangraula et al. (2021) demonstrate through the mhCACI framework that evidence-based psychological interventions can be systematically adapted and scaled to low-resource, community-level settings when contextualization is driven by the expressed experiences and explanatory models of the target population – precisely the kind of data our phenomenological inquiry has generated. Heim and Weise (2021) further emphasize that such cultural adaptation is not a cosmetic adjustment but a substantive process that shapes the delivery format, the role of community helpers, and the language and metaphors used to make psychosocial support meaningful. For Bede communities, this means that mobile MHPSS services should be co-designed with community sardars and bedenis, delivered in locally meaningful idioms (such as addressing “moner koshto” and “chinta” directly) and integrated with livelihood and governance support rather than offered as standalone clinic-based interventions. The participants’ expressed desire for modest, reliable support thus does not contradict MHPSS recommendations – it defines the conditions under which MHPSS can be both acceptable and effective for this population.

This study’s major strength lies in its qualitative phenomenological approach, which captures the lived experiences of displacement, loss and adaptation among river-nomadic communities with rich contextual depth. Conducting interviews during the monsoon season allowed participants’ narratives to reflect real-time challenges and emotional responses, increasing ecological validity. The inclusion of participants with diverse roles – such as sardars, sapures, bajigors and bedenis – enabled a broader understanding of how gender, occupation and leadership intersect in shaping psychosocial well-being. Methodological rigor was ensured through prolonged engagement, reflexive journaling and systematic thematic analysis guided by Braun and Clarke (2006, 2021), which enhanced credibility, dependability and confirmability.

However, several limitations must be acknowledged. The study’s small, context-specific sample limits the generalizability of findings beyond the selected Bede settlements in Tangail. Women’s participation was comparatively low, constraining gender-based insights into psychosocial distress and coping. The study relied entirely on self-reported experiences without clinical assessment tools such as the PHQ-9 or GAD-7, which could have provided measurable indicators of depression or anxiety. Moreover, the cross-sectional design prevents exploration of temporal changes in mental health outcomes across displacement cycles. Despite these limitations, the study offers valuable empirical insights for understanding the intersection of displacement, livelihood insecurity and psychosocial resilience among marginalized riverine populations in Bangladesh.

## Conclusion

This study explored how repeated environmental displacement affects the psychosocial well-being of river-nomadic Bede communities in Bangladesh. Through in-depth narratives, it revealed that mobility, while adaptive for survival, perpetuates instability, fatigue and anxiety. Environmental degradation, loss of traditional livelihoods and social exclusion collectively intensify psychological distress, yet strong community ties, faith-based practices and mutual support serve as critical sources of resilience. The findings emphasize that sustainable interventions must address both mental health and the ecological conditions driving displacement. Mobile, culturally sensitive MHPSS models, integrated with livelihood and governance support, can effectively respond to the needs of these mobile populations. Although limited in scale and scope, this research provides a grounded understanding of the mental health consequences of cyclical displacement and offers evidence to inform targeted, context-specific policy responses for marginalized riverine groups in Bangladesh.

## Data Availability

The data underlying this article consist of qualitative interviews and focus group discussion transcripts with members of highly identifiable and socially marginalized river-nomadic communities. In order to protect participant confidentiality and community privacy, full transcripts cannot be made publicly available. De-identified excerpts relevant to the analyses may be shared on reasonable request to the corresponding author, subject to approval by the Institutional Review Board and in line with participants’ consent and data protection requirements.

## Table 1. Long description

The table has four columns labeled Participant ID, Sex, Age in years, and Occupation or Social role. The first row lists Interview 1, Male, 70, Sardar community leader. The second row is Interview 2, Male, 32, Sapure snake charmer. The third row is Interview 3, Male, 60, Bajigor magician and healer. The fourth row is Interview 4, Male, 31, Sapure snake charmer. The fifth row is Interview 5, Male, 47, Sapure snake charmer. The sixth row is Interview 6, Female, 56, Sardar’s wife. The seventh row is Interview 7, Female, 25, Sapure bedeni snake charmer.

Navigate back to Table 1..

## Table 2. Long description

The header row contains Respondent I D, Sex, Age in years, and Occupation or Social role. The first row lists Respondent 1, Male, 35, Sapure. The second row lists Respondent 2, Male, 42, Sapure. The third row lists Respondent 3, Male, 46, Sapure. The fourth row lists Respondent 4, Male, 58, Bajigor. The fifth row lists Respondent 5, Male, 22, Unemployed. All respondents are male, ages range from 22 to 58. Three respondents are Sapure, one is Bajigor, and one is unemployed.

Navigate back to Table 2..

## Table 3. Long description

The table has three columns: Theme, Core insight, and Psychosocial implication. From top to bottom, the themes are: 1. Income-driven relocation, with the insight that mobility is a planned survival strategy guided by shifting livelihood opportunities, and the implication that economic precarity fosters adaptive decision-making but sustains chronic instability. 2. Environmental hazards, with the insight that floods, erosion, and storms force rapid displacement and recurrent loss, and the implication that environmental stress translates into cumulative anxiety and trauma. 3. Physical and emotional toll, with the insight that each relocation imposes acute physical exhaustion and emotional distress expressed through culturally specific idioms of suffering, and the implication that cumulative distress requires recognition of local idioms and culturally sensitive screening beyond Western clinical instruments. 4. Social negotiation and reputation, with the insight that settling safely depends on negotiating acceptance with locals and maintaining social reputation, and the implication that social capital becomes a protective but fragile buffer against exclusion. 5. Culturally rooted coping and resilience, with the insight that communities actively cope through faith-based practices, communal mutual aid, and adaptive meaning-making rooted in cultural explanatory models, and the implication that indigenous resilience strategies provide an essential foundation for culturally adapted M H P S S co-design and community-based delivery. 6. Aspirations for stability, with the insight that participants desire secure housing, steady income, and institutional recognition, and the implication that this highlights unmet policy needs and the psychosocial cost of systemic neglect.

Navigate back to Table 3..
