# Supplementary material for: The consequences of migration and displacement for mental health: A qualitative study of river nomadic communities
Source: Glob Ment Health (Camb). 2026 May 26;13:e120. doi: 10.1017/gmh.2026.10230 (PMC13279963; doi:10.1017/gmh.2026.10230)
Supplement: Peer review history

# Reviewer report: Review: The consequences of migration and displacement for mental health: A qualitative study of river nomadic communities — R0/PR2

**DOI:** 10.1017/gmh.2026.10230.pr2

**Authors:** Sara Akram

**Affiliations:** Sun Yat-Sen University, China

This manuscript offers a compelling and original qualitative examination of the psychosocial consequences of recurrent environmental displacement among river-nomadic (Bede) communities in Bangladesh. The study addresses a critical gap in global mental health and climate migration scholarship by focusing on a highly marginalized and understudied population whose mobility is shaped by ecological change and structural exclusion.

The phenomenological research design is well aligned with the study objectives, and the use of in-depth interviews and a focus group discussion provides rich and nuanced empirical material. Data collection during the monsoon season strengthens the contextual validity of the findings, and the application of Braun and Clarke’s thematic analysis framework is methodologically sound and transparently executed. The six themes identified are coherent, analytically robust, and well supported by participant narratives.

A key strength of the manuscript is its conceptual framing of displacement as a continuous psychosocial exposure rather than a single event. The balanced discussion of distress and resilience, including faith-based coping, communal solidarity, and adaptive strategies, adds significant depth to existing literature on climate-induced displacement and mental health. The conclusions are well grounded in the findings and offer clear, actionable implications for mobile, culturally sensitive mental health and psychosocial support interventions.

The manuscript is clearly written, theoretically informed, and makes a strong contribution to the field. It aligns well with the scope and aims of Cambridge Prisms: Global Mental Health. I recommend this paper for acceptance, as it meets high scholarly standards and offers valuable insights for researchers, practitioners, and policymakers.

# Reviewer report: Review: The consequences of migration and displacement for mental health: A qualitative study of river nomadic communities — R0/PR3

**DOI:** 10.1017/gmh.2026.10230.pr3

Thank you for the opportunity to review this paper on the impact of climate change–related displacement on mental health among affected individuals in Bangladesh. I believe this is a timely and important topic, given the increasing physical and mental health risks associated with climate change. This paper reports qualitative findings from in-depth interviews and focus group discussions analysed using a phenomenological approach.

Introduction

1. The introduction is well written, with a clear and logical sequence of arguments that leads to the main objectives of the study. My only suggestion is to consider whether there are existing studies reporting the prevalence of mental health problems among river-nomadic communities in Bangladesh or comparable settings. While the authors note that prior research has largely been quantitative, some of these studies may include estimates of mental health burden that could strengthen the rationale for the current study by highlighting the urgency of understanding lived experiences of forced migration.

Methods

I applaud the researchers for clearly describing their recruitment process and the care taken during recruitment.

1. Given the constructivist nature of the analysis, the authors should elaborate on their positionality and reflexivity.

2. Who collected the data?

3. What was the rationale for using focus group discussions rather than in-depth interviews?

4. Who conducted the data analysis, and how did the authors ensure iteration and reflexivity throughout the analytic process?

5. Over the years, Braun and Clarke’s thematic analysis has often been treated as a single, unified method without clearly articulating its underlying epistemological and methodological assumptions. I strongly recommend that the authors report their thematic analysis process in line with recent guidance by Braun and Clarke (Braun, V., & Clarke, V. (2023). Toward good practice in thematic analysis: Avoiding common problems and be (com) ing a knowing researcher. International journal of transgender health, 24(1), 1-6.)). I also suggest that the authors clearly specify which approach to thematic analysis they adopted (e.g., coding reliability, codebook, or reflexive thematic analysis), following Braun, V., & Clarke, V. (2021). One size fits all? What counts as quality practice in (reflexive) thematic analysis?. Qualitative research in psychology, 18(3), 328-352

Findings

1. I suggest revising the sentence “Thematic analysis identified…” to “We identified six interconnected themes.”

2. In addition, different participant quotes should be used under different themes. Currently, the following quote is repeated:

“If people remember us from before and we helped them, they welcome us again; it helps us earn safely and consistently.”

3. It is unclear how the theme of fatigue and anxiety is distinct from physical strain, mental health distress, and resilience strategies, which is a very broad theme. Similar quotes appear under both themes. It may be worth considering merging fatigue and anxiety into the latter theme and potentially separating physical and mental health sequelae.

4. I appreciate how succinctly the authors summarise their main findings; however, the table presenting these findings should be included in the Results section rather than the Discussion.

Discussion

1. Compared to other sections of the manuscript, the Discussion is relatively narrow. As a reader, I would like to see more emphasis on how these findings extend or advance the existing literature, rather than primarily reiterating what has already been reported in previous studies.

2. The practice and policy implications are also limited. In particular, the paragraph on Page 15 (Lines 11–16) does not follow logically. It is unclear how participants’ expressed desire for modest and predictable support aligns with the recommendations around task-sharing and community-based interventions.

Finally, the expressed desire for modest, predictable supports (e.g., secure plots, livelihood facilitation, access to medicine, and schooling) aligns well with recommendations for culturally adapted, mobility-sensitive mental health and psychosocial support (MHPSS) in low- and middle-income countries. These approaches favour community-based delivery, task-sharing, and integration with social protection systems where clinic-based models are inaccessible to mobile populations (Sheath et al., 2020; Haque et al., 2022; Hayes et al., 2018).

# Reviewer report: Review: The consequences of migration and displacement for mental health: A qualitative study of river nomadic communities — R0/PR4

**DOI:** 10.1017/gmh.2026.10230.pr4

Review comments: GMH-2025-0410

ABSTRACT

Page-2; line 36: The authors mention themes “emerged.” I encourage the authors to edit terminology (“emerged”), in the abstract and in the methods, to something more aligned with what Braun and Clarke’s reflexive thematic analysis (TA) method claims, that is, that themes do not emerge from the data, but rather are constructed by researchers using the reflexive TA 6 step process. This Editorial from Qualitative Social Work (cited below) summarizes the methodological progression of Braun and Clarke’s stance of themes being constructed rather than emerging, and contains the citations to original articles/books by Braun and Clarke on this common misappropriation of reflexive TA. Please see excerpt from the editorial referenced below.

“The change reflects Braun and Clarke’s approach to thematic analysis, making clear that themes do not emerge from the data, a sentence we often see in articles submitted to our journal. Instead, the researcher actively constructs each of the themes through their engagement with the material.”

Morriss, L. (2024). Themes do not emerge. An editor’s reflections on the use of Braun and Clarke’s thematic analysis. Qualitative Social Work, 23(5), 745-749. https://doi.org/10.1177/14733250241277355 (Original work published 2024)

INTRODUCTION

Page-4, line8: multisectoral – perhaps “multidimensional” is better suited?

Page-5, lines 19-20: idioms of distress – Recommend citing Nichter 1981 or subsequent works here.

Page-5, line 33: explanatory model – Recommend citing Kleinman’s “patients and healers in the context of culture” here.

METHODS

I request the authors to complete and submit the COREQ statement for the study as a supplemental file, and make the COREQ specific necessary changes to the manuscript.

Specifically, could you provide more detail about your codes and codebook?

Page-7, line 26: sample size – I feel your sample size is more than sufficient for the study design, and justified well. You can consider citing Guest et al 2006, which endorse saturation at 12 qualitative interviews, as evidence-driven methodological support for your sample; or Materud et al. 2016, for a more conceptually driven justification for the sample size. This is at your discretion; please only add if helpful.

Guest, G., Bunce, A., & Johnson, L. (2006). How Many Interviews Are Enough?: An Experiment with Data Saturation and Variability. Field Methods, 18(1), 59-82. https://doi.org/10.1177/1525822X05279903 (Original work published 2006)

Malterud, K., Siersma, V. D., & Guassora, A. D. (2016). Sample Size in Qualitative Interview Studies: Guided by Information Power. Qualitative health research, 26(13), 1753–1760. https://doi.org/10.1177/1049732315617444

RESULTS:

Most of the results are well-constructed. I do, however, find that the descriptions and experiences of mental health, stress, psychological distress, etc. to be superficial and rushed. Could more detail (quotes, perhaps in a Table, if word limit is a concern) and description be added? Did you identify idioms of distress and explanatory models (Kleinman’s categories include: label/name; pathophysiology or symptoms; severity; fears, treatment beliefs/attitudes), as the Introduction/Methods sets up the reader to expect these in the Results – could those be reported on with more detail?

These issues resurface in the DISCUSSION:

On page-14, line 30, it is claimed the study findings align w/ research that finds elevated symptoms of depression and anxiety – however the shallow presentation of psychopathology in the results make this claim weak.

Additionally, on page-14, line45-46 there is a claim that the current study’s findings (in narratives “discussing shifting river courses and disappearing aquatic livelihoods”) are supportive of literature on anticipatory anxiety, and grief linked to ecosystem loss – this is not supported well by the data presented in the results. Please either include this data or consider removing this claim.

Page-15, line 10-16 - I am struggling to see how “the expressed desire for modest, predictable supports (secure plots, livelihood facilitation, medicine, schooling)” align with or can inform MHPSS cultural adaptation or delivery, as you state – those are structural interventions and not MHPSS interventions. I think more critical engagement needs to happen here to demonstrate how the study findings connect with MHPSS adaptation and delivery, especially as that is one of your explicitly stated study objectives (page-6, line 23-24). You can consider the following articles in shaping this narrative:

Heim, E., & Kohrt, B. A. (2019). Cultural Adaptation of Scalable Psychological Interventions: A New Conceptual Framework. Clinical Psychology in Europe, 1(4), Article e2555. https://doi.org/10.32872/cpe.v1i4.37679

Sangraula, M., Kohrt, B. A., Ghimire, R., Shrestha, P., Luitel, N. P., Van’t Hof, E., Dawson, K., & Jordans, M. J. D. (2021). Development of the mental health cultural adaptation and contextualization for implementation (mhCACI) procedure: a systematic framework to prepare evidence-based psychological interventions for scaling. Global mental health (Cambridge, England), 8, e6. https://doi.org/10.1017/gmh.2021.5

Heim, E., & Weise, C. (2021). Special Issue Editorial: Cultural Adaption of Psychological Interventions. Clinical psychology in Europe, 3(Spec Issue), e7627. https://doi.org/10.32872/cpe.7627

# Editor report: Recommendation: The consequences of migration and displacement for mental health: A qualitative study of river nomadic communities — R0/PR5

**DOI:** 10.1017/gmh.2026.10230.pr5

**Affiliations:** The George Institute for Global Health India, India

Please revise and address the points raised by the reviewers.

# Editor report: Decision: The consequences of migration and displacement for mental health: A qualitative study of river nomadic communities — R0/PR6

**DOI:** 10.1017/gmh.2026.10230.pr6

**Affiliations:** London School of Hygiene & Tropical Medicine, United Kingdom of Great Britain and Northern Ireland

No accompanying comment.

# Reviewer report: Review: The consequences of migration and displacement for mental health: A qualitative study of river nomadic communities — R1/PR8

**DOI:** 10.1017/gmh.2026.10230.pr8

I thank the reviewers for their engagement with the review comments and the changes made to the manuscript. I think the manuscript is significantly strengthened as a result.

Two quick points:

1. just pointing out one discrepancy - in the first page of the proof shared for peer review of the revised manuscript (likely drawing from the metadata submitted to the journal’s forms), the “themes emerged” language is still present although I see authors have revised this in the body.

2. The MHPSS paragraph is missing in both track changes and clean manuscripts - pls check: Change made: Discussion MHPSS paragraph fully rewritten using Heim & Kohrt (2019), Sangraula et al. (2021), and Heim & Weise (2021). Structural supports distinguished from MHPSS interventions. All three references added to reference list.

# Reviewer report: Review: The consequences of migration and displacement for mental health: A qualitative study of river nomadic communities — R1/PR9

**DOI:** 10.1017/gmh.2026.10230.pr9

**Authors:** Sara Akram

**Affiliations:** Sun Yat-Sen University, China

Thank you for the opportunity to review your revised manuscript. This is a compelling qualitative study that sheds light on an under-researched population—the Bede river nomads—and the complex psychosocial toll of recurrent environmental displacement.

The phenomenological approach is appropriate, and the six themes you constructed are well supported by rich participant accounts. The integration of NVivo 14 with Braun and Clarke’s reflexive thematic analysis is clearly described. I particularly appreciate how you highlight both chronic distress and the community’s culturally rooted resilience, offering a balanced perspective.

The revisions have strengthened the clarity of your methods and the practical implications for mobile MHPSS services. I have no further concerns and recommend acceptance. This work makes a valuable contribution to the growing literature on climate-related migration and mental health.

Well done.

# Reviewer report: Review: The consequences of migration and displacement for mental health: A qualitative study of river nomadic communities — R1/PR10

**DOI:** 10.1017/gmh.2026.10230.pr10

The authors have addressed most of my comments. There is still one that I don’t think the authors fully engaged with. Themes 3 and 5 are still very much the same. Their opening sentence is even the same, but paraphrased differently. The content is the same except for the resilience strategies mentioned in Theme 5. I strongly recommend that the authors revise these two themes and combine the physical and emotional toll/stress into one theme, and copign/resilience strategies into another.

# Editor report: Recommendation: The consequences of migration and displacement for mental health: A qualitative study of river nomadic communities — R1/PR11

**DOI:** 10.1017/gmh.2026.10230.pr11

**Affiliations:** The George Institute for Global Health India, India

Pl address the remaining minor comments and resubmit.

# Editor report: Decision: The consequences of migration and displacement for mental health: A qualitative study of river nomadic communities — R1/PR12

**DOI:** 10.1017/gmh.2026.10230.pr12

**Affiliations:** London School of Hygiene & Tropical Medicine, United Kingdom of Great Britain and Northern Ireland

No accompanying comment.

# Reviewer report: Review: The consequences of migration and displacement for mental health: A qualitative study of river nomadic communities — R2/PR14

**DOI:** 10.1017/gmh.2026.10230.pr14

n/a

# Reviewer report: Review: The consequences of migration and displacement for mental health: A qualitative study of river nomadic communities — R2/PR15

**DOI:** 10.1017/gmh.2026.10230.pr15

**Authors:** Sara Akram

**Affiliations:** Sun Yat-Sen University, China

This study provides a vital and deeply empathetic exploration of the mental health challenges facing the river-nomadic Bede communities in Bangladesh. By reframing displacement as a continuous, cyclical exposure rather than a single event, the authors offer a sophisticated understanding of how environmental hazards, economic precarity, and social marginalization intersect to create chronic psychological distress. The research is particularly commendable for its methodological authenticity, capturing real-time narratives during the monsoon season and highlighting the community’s own “culturally rooted” resilience strategies. These insights offer a crucial roadmap for moving away from rigid, clinic-based models toward mobile, community-based support systems. Overall, this is a high-quality, rigorous piece of qualitative scholarship that significantly advances the discourse on climate-related migration and global mental health.

# Reviewer report: Review: The consequences of migration and displacement for mental health: A qualitative study of river nomadic communities — R2/PR16

**DOI:** 10.1017/gmh.2026.10230.pr16

No further comments. Congratulations to the authors!

# Editor report: Recommendation: The consequences of migration and displacement for mental health: A qualitative study of river nomadic communities — R2/PR17

**DOI:** 10.1017/gmh.2026.10230.pr17

**Affiliations:** The George Institute for Global Health India, India

No accompanying comment.

# Editor report: Decision: The consequences of migration and displacement for mental health: A qualitative study of river nomadic communities — R2/PR18

**DOI:** 10.1017/gmh.2026.10230.pr18

**Affiliations:** London School of Hygiene & Tropical Medicine, United Kingdom of Great Britain and Northern Ireland

No accompanying comment.
